# Genetic Differentiation, Diversity, and Drug Susceptibility of *Candida krusei*

**DOI:** 10.3389/fmicb.2018.02717

**Published:** 2018-11-20

**Authors:** Jie Gong, Meng Xiao, He Wang, Timothy Kudinha, Yu Wang, Fei Zhao, Weiwei Wu, Lihua He, Ying-Chun Xu, Jianzhong Zhang

**Affiliations:** ^1^State Key Laboratory of Infectious Disease Prevention and Control, Collaborative Innovation Center for Diagnosis and Treatment of Infectious Diseases, National Institute for Communicable Disease Control and Prevention, Chinese Center for Disease Control and Prevention, Beijing, China; ^2^Department of Clinical Laboratory, Peking Union Medical College Hospital, Chinese Academy of Medical Sciences, Beijing, China; ^3^Beijing Key Laboratory for Mechanisms Research and Precision Diagnosis of Invasive Fungal Diseases, Beijing, China; ^4^School of Biomedical Science, Charles Sturt University, Orange, NSW, Australia; ^5^Central West Pathology Laboratory, Orange, NSW, Australia; ^6^Key Laboratory of Wildlife Biotechnology, Conservation and Utilization of Zhejiang Province, Zhejiang Normal University, Jinhua, China; ^7^Department of Dermatology, Hainan Provincial Center for Skin Disease and STI Control, Haikou, China

**Keywords:** *Candida krusei*, invasive candidiasis, genetic differentiation, genetic diversity, microsatellites, drug susceptibility

## Abstract

*Candida krusei* is a notable pathogenic fungus that causes invasive candidiasis, mainly due to its natural resistance to fluconazole. However, to date, there is limited research on the genetic population features of *C. krusei*. We developed a set of microsatellite markers for this organism, with a cumulative discriminatory power of 1,000. Using these microsatellite loci, 48 independent *C. krusei* strains of clearly known the sources, were analyzed. Furthermore, susceptibility to 9 antifungal agents was determined for each strain, by the Clinical and Laboratory Standards Institute broth microdilution method. Population structure analyses revealed that *C. krusei* could be separated into two clusters. The cluster with the higher genetic diversity had wider MIC ranges for six antifungal agents. Furthermore, the highest MIC values of the six antifungal agents belonged to the cluster with higher genetic diversity. The higher genetic diversity cluster might have a better adaptive capacity when *C. krusei* is under selection pressure from antifungal agents, and thus is more likely to develop drug resistance.

## Introduction

Invasive candidiasis is the most common fungal disease among hospitalized patients, and affects more than 250,000 people worldwide annually, with more than 50,000 deaths reported (Kullberg and Arendrup, 2015). In the *Candida* genus, *Candida krusei* attracts much medical attention because it is intrinsically resistant to fluconazole (Akova et al., 1991; Schuster et al., 2013). In addition, *C. krusei* exhibits resistance to other antifungal drugs such as voriconazole, echinocandins, and amphotericin B (Fukuoka et al., 2003; Hakki et al., 2006; Pfaller et al., 2008). It has been known for some time that mutations in *ERG11* and *FKS 1* genes are the major mechanisms responsible for azole- and echinocandin-resistance in *Candida* species, including *C. krusei* (Jensen et al., 2014; Forastiero et al., 2015; Feng et al., 2016; Perlin et al., 2017). In addition, antifungal resistance can be acquired by over-expression of efflux pump e.g., Abc1p (Lamping et al., 2009; Ricardo et al., 2014). However, there have been some *C. krusei* antifungal resistant phenotypes, including resistance to azoles other than fluconazole and to enchinocandins e.g., caspofungin, that cannot be explained by currently known mechanisms of resistance (Hakki et al., 2006; Whaley et al., 2017).

From an evolutionary perspective, drug resistance in a microorganism is part of the adaptive evolutionary response of a species to environmental pressures (Salmond and Welch, 2008). Nowadays, the environmental pressure of antifungal drugs comes not only from the use of clinical drugs, but also from the use of agricultural drugs (Sanglard, 2016).

The adaptive capacity is usually related to the level of genetic diversity. From the points of molecular ecology, genetic diversity can allow species or populations to adapt quickly to changing environment conditions and different habitats (Freeland et al., 2011). Similarly, from the perspective of conservation genetics, genetic diversity allows species or populations to tolerate a wider range of environmental changes, including bacteria, fungi and so on. Also, genetic diversity is helpful to maintain the evolutionary vigor (Frankham et al., 2010). In general, a higher genetic diversity enables the organism to respond better to new selection pressures (McDonald and Linde, 2002). When there was a selection pressure for exogenous antifungal agents, the more genetic diversity the fungal populations had, the higher the probability of survival. In other words, antifungal agents were the directional selection factors from Darwin's theory of Evolution. If the genetic diversity of the fungal population was high, there might be some individual death under the pressure of drug selection, but some individuals carrying different genes would survive. Therefore, it is reasonable to hypothesize that microbial populations with higher genetic diversity are more likely to develop antimicrobial drug resistance.

However, to our best knowledge, no research targeting the genetic population features of *C. krusei* has been carried out to date. This is partly due to the lack of a flexible molecular typing method. Therefore, in this study, we (1) developed a novel set of microsatellite markers for molecular typing and population genetic analysis of *C. krusei*; (2) used the developed assay to type 48 multicenter collected *C. krusei* clinical strains; and (3) analyzed the correlation between genetic diversity and drug susceptibility among the studied strains.

## Materials and methods

### Ethics statement

This study was reviewed and approved by the ethics committee of the National Institute for Communicable Disease Control and Prevention, Chinese CDC. Written informed consent was obtained from patients for use of the samples in research.

### Isolate collection and identification

**Figure 1:**
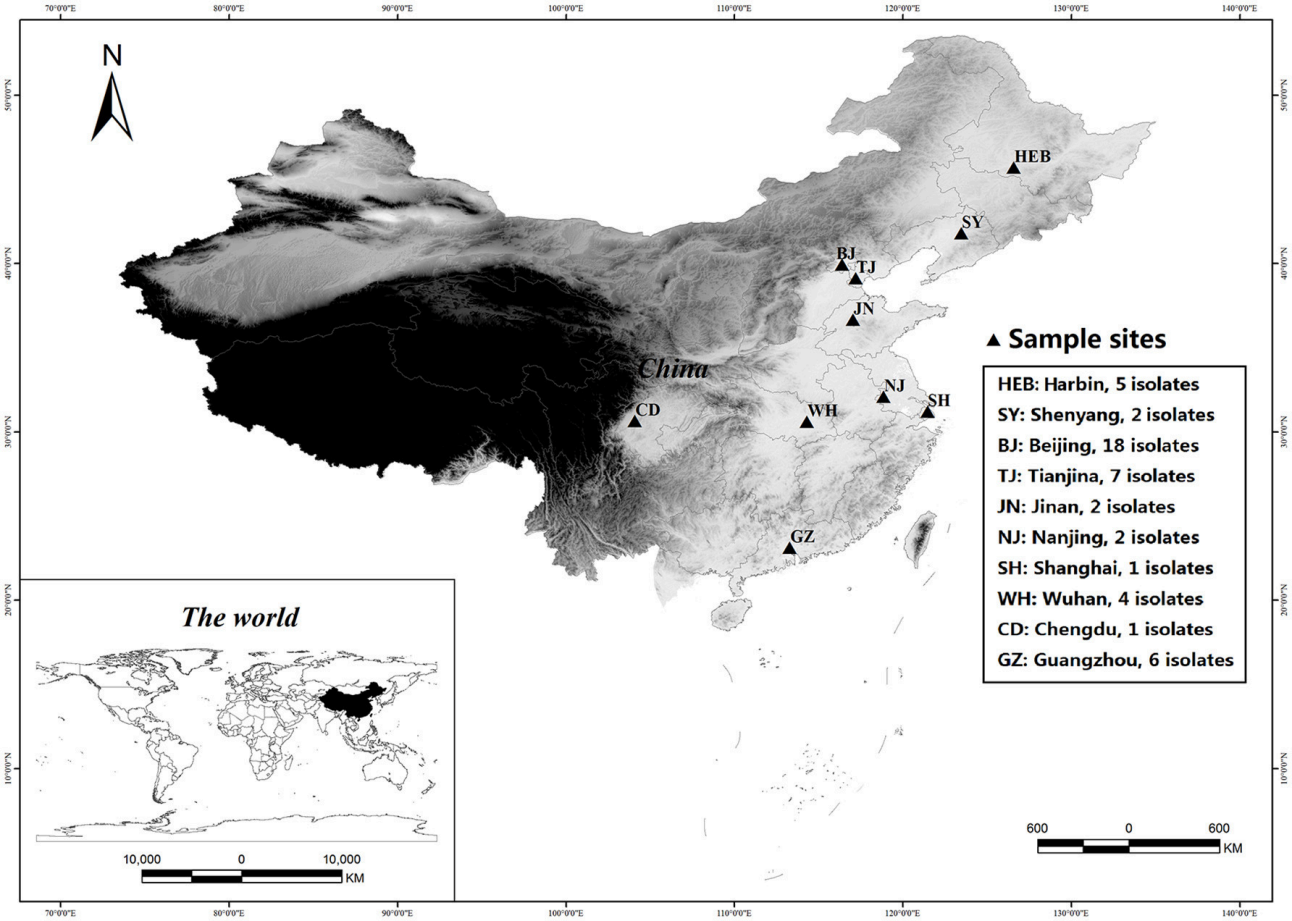
Geographical locations of sampled *Candida krusei* populations in China. The figure is generated by ArcGIS Desktop (version 9.3, ESRI, Redlands, USA).

A total of 48 *C. krusei* isolates, each from a single patient, were collected from 15 hospitals distributed in 10 cities across China during the period 2009–2012, as part of the national surveillance program for invasive fungal infections (the CHIF-NET study, Figure 1 and Supplemental A). The isolates were stored at −80°C until use at Peking Union Medical College Hospital, Beijing, China (PUMCH). Before testing, the isolates were inoculated on CHROMagar™ *Candida* medium (Difco Laboratories, Detroit, MI, USA) and incubated at 37°C for 24 h. Species identification of the isolates was confirmed by matrix-assisted laser desorption/ionization-time of flight mass spectrometry (MALDI-TOF MS, Vitek MS, bioMérieux, Marcy-l'E'toile, France) as per manufacturer's instructions, and by sequence analysis of their rDNA internal transcribed spacer (ITS) regions (Wang et al., 2012). The identities of all the isolates was confirmed by sequencing.

### DNA extraction, microsatellite development, and genotyping

All the fungal isolates were grown on potato dextrose agar at 37°C for 24 h. DNA extraction was performed using a QIAamp DNA Mini Kit (Qiagen, Hilden, Germany).

The software SciRoKo was used to identify microsatellites in the *C. krusei* genome (GenBank assembly accession: GCA_001983325.1, Kofler et al., 2007; Cuomo et al., 2017). Primers were designed using Primer premier 5.0 (PREMIER Biosoft International, Palo Alto, CA, USA) in regions flanking microsatellite loci, and annealing temperatures were optimized with a gradient PCR. Polymorphic microsatellite loci were selected for molecular typing and population genetic analysis of *C. krusei*. There were two criteria used for selection of microsatellite loci: first, the locus had to have a relatively high genetic polymorphism (the number of alleles was >3); second, the locus could be amplified relatively stable. The microsatellite loci would be abandoned if the loci was not amplified in more than two strains.

For the 33 selected microsatellite loci (Table 1), PCR was performed on 48 clinical isolates. Amplification was carried out using a *Taq* polymerase kit (Takara, Dalian, China). Each of the amplification reactions was composed of 1 × PCR buffer, 0.2 μM dNTP, 0.5 U *Taq* polymerase, 0.2 μM each primer, and 2 μl genomic DNA (20–50 ng/μl). The thermocycler conditions were as follows: initial denaturation at 95°C for 5 min, followed by 35 cycles of denaturation at 94°C for 30 s, annealing at an optimized primer-specific annealing temperature for 30 s (Table 1), extension at 72°C for 30 s and final extension at 72°C for 10 min. The primers for these selected loci were fluorescently labeled with 6-carboxy-fluorescein (6-FAM). Allele length was determined by migration of PCR products on an ABI 3,700 automated capillary DNA sequencer (Applied Biosystems). Allele sizes were assigned with GeneMapper software (version 3.7) according to an internal size standard (LIZ 500, Applied Biosystems).

**Table 1:** Characterization of *Candida krusei* microsatellite loci.

**Locus**	**Primer sequences (5′−3′)**	**Ta^a^ (°C)**	**Repeat type**	**Size range (bp)**	**No. of alleles**	**PIC^b^**	**DP^c^**	**NCBI accession**
Cakr001	ACGGACCCACAACATCAAC	56	(AT)_11_	381–394	7	0.663	0.701	MH079517
	GAAGGGGAGGTAAGGAGA						
Cakr002	GTAGAACCCGTATGAGGAC	52	(TA)_13_	293–303	6	0.708	0.750	MH079518
	GTAGAACCCGTATGAGGAC						
Cakr003	CATATCACTATGACATTCCA	52	(TCT)_11_	284–272	5	0.604	0.663	MH079519
	TCCATCTATCCGCAACAAG						
Cakr004	AAGGACGGTGCTTTCAATC	59	(TAT)_12_	365–401	11	0.739	0.768	MH079520
	TTTACGACGGTTTCCAGTG						
Cakr005	CAGTCAACTCGCCCTCCCT	62	(AAT)_17_	311–362	16	0.867	0.878	MH079521
	CAGTGTTTGTGCCTGTGCC						
Cakr006	TAGTTTCGGGACTCTGTAT	56	(TC)_12_	362–370	5	0.551	0.621	MH079522
	TCACGTTGTAACCGAGGTA						
Cakr007	GTAGGCGGCGAAGGAAGAT	59	(TCA)_11_T(CAT)_9_	174–261	8	0.803	0.826	MH079523
	TAACAACAGCAACCGAAAG						
Cakr008	AGCACCCTGAAAACTCTAC	52	(TA)_12_	245–257	6	0.665	0.716	MH079524
	ATCTACAAGCGTTCTAAAT						
Cakr009	AGTATCCGAGTCTGGTTTA	56	(AGA)_10_	223–256	9	0.561	0.586	MH079525
	GGTAGGCTTCTCAGTTTTA						
Cakr010	TTGTCGGATTTGTGGTAAG	54	(GAA)_10_	278–323	7	0.586	0.640	MH079526
	CATCGTCAGCATTTTCACT						
Cakr011	AGTTGGAGTTGTGGGGAGA	62	(CTTGAC)_13_	357–453	13	0.822	0.837	MH079527
	GAGACGGGTTACCAAGGAT						
Cakr012	GCAATGTCGGAAATGAACTAG	59	(AT)_11_	346–356	6	0.711	0.750	MH079528
	AAGGACGAGAACAGCAAGAA						
Cakr013	TTGGTAAGTTGGTGGGACG	59	(AT)_10_	246–252	4	0.335	0.356	MH079529
	ACATTGGGAAGCGGAAGAA						
Cakr014	CCAAGGCAATGTCAGGAAC	59	(TG)_18_	178–190	6	0.714	0.751	MH079530
	TTGTAGAGGACGGAATCTC						
Cakr015	CTCCTGGCATTGCCGTTAT	59	(AC)_11_	295–305	5	0.696	0.742	MH079531
	AAGCGGGAAGTTGTAGATT						
Cakr016	TAACTAAACACGTTTACCA	54	(AT)_10_	193–199	4	0.313	0.350	MH079532
	TTTAGGATTTGCTCTTTCA						
Cakr017	GACAAGAAATGCGGGAACC	59	(AT)_10_	284–314	7	0.661	0.703	MH079533
	GGCGATGACAGCGATAGTG						
Cakr018	CATCGGAGGCTGGTAAATA	59	(TA)11	284–294	6	0.604	0.658	MH079534
	TACGGAGTCGTCCCTTGAT						
Cakr019	CGATTTCTAGTGGTGTTAGT	54	(TCA)_11_	225–264	11	0.695	0.717	MH079535
	ATACTCTTAGCCCTGATACA						
Cakr020	TCCACAAACACCGAAACACT	59	(AAC)_11_	275–311	9	0.735	0.771	MH079536
	ATAGACATGGGCCAAATGAG						
Cakr021	AGACCAACAGAGGAGGGACA	56	(TA)_11_	343–365	9	0.789	0.814	MH079537
	ACGATAAATGATTTTCAAGC						
Cakr022	CGTTTATTCATGCCTTCCTC	59	(AT)_10_	310–316	4	0.463	0.539	MH079538
	TAATGGTAATGCGGCTGATG						
Cakr023	GTTAGTGGCACCAAAGAGGA	59	(TA)_11_	267–286	9	0.638	0.690	MH079539
	GATGATGACTTCAAGGACGG						
Cakr024	CTGACACTACTATTTATTGGGATG	56	(AAC)_10_	398–425	9	0.539	0.565	MH079540
	TGTTTGGTATGATATTCAATGTGC						
Cakr025	AAACAGGGAAAGAATCATAA	54	(AC)_10_	263–321	11	0.683	0.728	MH079541
	TGTATTGTAGCACCTAAAGC						
Cakr026	GGCATGGTTTGTCGTCGTGT	59	(TA)_10_	294–314	11	0.699	0.720	MH079542
	GAGGGGACTTGGCAGAGGGA						
Cakr027	CGAAGTTTTGGTTTCTTTAA	54	(AT)_10_	270–286	8	0.663	0.695	MH079543
	CATTCACCAATCCTTGTTAC						
Cakr028	TTGGAAAGCAACTTAGAGTC	56	(AT)_10_	248–254	4	0.652	0.708	MH079544
	TAGGTCTAAAGCAGAACGAG						
Cakr029	GTCTAGTCTCGCAATACCTC	54	(CA)_10_(CT)_17_	246–286	14	0.801	0.822	MH079545
	CTCTTTGGATTTCCTTTTAT						
Cakr030	AAACTCGGAATCTCCAAACG	59	(CTT)_11_	147–168	8	0.557	0.581	MH079546
	GTACCACTGGGCGAAAACAA						
Cakr031	CCTTGTTGGTAATAGTTTTC	52	(TCT)_10_	347–392	10	0.636	0.659	MH079547
	CTAACGAGGAAGTTGTATGT						
Cakr032	TGCGTTTCTCAGAGGCTGTT	56	(TC)_10_	193–203	5	0.488	0.550	MH079548
	GTGGGGATAGGTGTTTGGTG						
Cakr033	GCGCTTCAGTGGTAGTCATA	56	(CAA)_11_	265–289	6	0.701	0.739	MH079549
	TTCCACAAACTTGAACTCGTC						
Mean	–	–	–	–	7.848	0.647	–	–
Overall	–	–	–	–	–	–	1.000	–

a Annealing temperature;

b Polymorphic information content;

c *discriminatory power*.

### Antifungal susceptibility testing

The *in vitro* susceptibility to nine antifungal drugs- fluconazole, voriconazole, itraconazole, posaconazole, caspofungin, micafungin, anidulafungin, amphotericin B, and 5-flucytosine, was determinedfor 48 isolates using the Clinical and Laboratory Standards Institute (CLSI) broth microdilution method^22^. Minimum inhibitory concentration (MIC) results for fluconazole, voriconazole, caspofungin, micafungin, and anidulafungin, were interpreted using clinical breakpoints in accordance with the CLSI guidelines (CLSI, 2017), and those for itraconazole, posaconazole, amphotericin B, and 5-flucytosine, were interpreted using epidemiological cut-off values (Xiao et al., 2014). The quality control strains used were *C.krusei* ATCC 6,258 and *Candida parapsilosis* ATCC 22,019.

### Data analysis

Deviation from Hardy-Weinberg equilibrium was computed using GENEPOP version 4 (Rousset, 2008). Hardy-Weinberg equilibrium was tested using the score test for heterozygote deficiency and the significance was addressed by a Markov Chain algorithm (Markov chain parameters: dememorization number = 2,000, number of batches = 250, number of iterations per batch = 2,000).

The discriminatory power of markers was calculated according to the method of Hunter and Gaston (1988). Number of alleles (n_A_), effective number of alleles (n_e_), Shannon's Information Index (I), and Nei's unbiased gene diversity (H_S_), were calculated using GENALEX 6.5 (Peakall and Smouse, 2012). Allelic Richness (AR) was calculated by FSTAT 2.9.3 (Goudet, 1995). Ne, I, HS, and AR were used to measure genetic variability of populations.

Population composition was inferred for the *C. krusei* isolates using the program Structure 2.3 (Pritchard et al., 2000), which estimates the log probability of the data for each value of K (number of clusters or populations). A series of independent runs were performed by using K from 1 to 12 populations, a burn-in of 100,000 Markov chain Monte Carlo (MCMC) iterations, and a data collection period of 100,000 MCMC iterations. Each simulation of K was replicated 10 times. The method of Evanno et al. (2005) was used to estimate the most likely K given the data with Structure Harvester (Earl, 2012). The level of genetic differentiation at microsatellite loci among clusters was estimated as F_ST_, which is simply a measure of how genetically similar populations are to one another. F_ST_ was calculated using Arlequin 3.5 (Excoffier and Lischer, 2010). Principal coordinates analysis (PCoA) of F_ST_ value among clusters was calculated using GenAlEx (Peakall and Smouse, 2012).

For antifungal susceptibility results, MIC_50_, MIC_90_, and geometric mean (GM) MIC values were calculated using WHONET software (version 5.6, WHO Collaborating Center for Surveillance of Antimicrobial Resistance, Boston, USA).

## Results

### Microsatellite loci of *C. krusei*

Based on the genome of *C. krusei*, a total of 200 microsatellite loci were identified, and primers were designed (data not shown). Of these microsatellite loci, 33 polymorphic microsatellite loci (Cakr 001–Cakr 033) could be stably amplified in all *C. krusei* isolates (Table 1). The cumulative discriminatory power of the 33 loci was 1.000. If only 8 polymorphic sites with the highest polymorphism were selected (Cakr004, Cakr005, Cakr011, Cakr019, Cakr025, Cakr026, Cakr029, Cakr031), it was found that the cumulative discriminatory power would still be 1.000. This might mean that the molecular typing of strains could be achieved effectively by using only these 8 microsatellite loci. All loci showed significant deviation from Hardy-Weinberg equilibrium (*P* < 0.05).

In addition, it must be noted that many isolates were heterozygote (Supplemental A).

### Genetic differentiation and diversity

**Figure 2:**
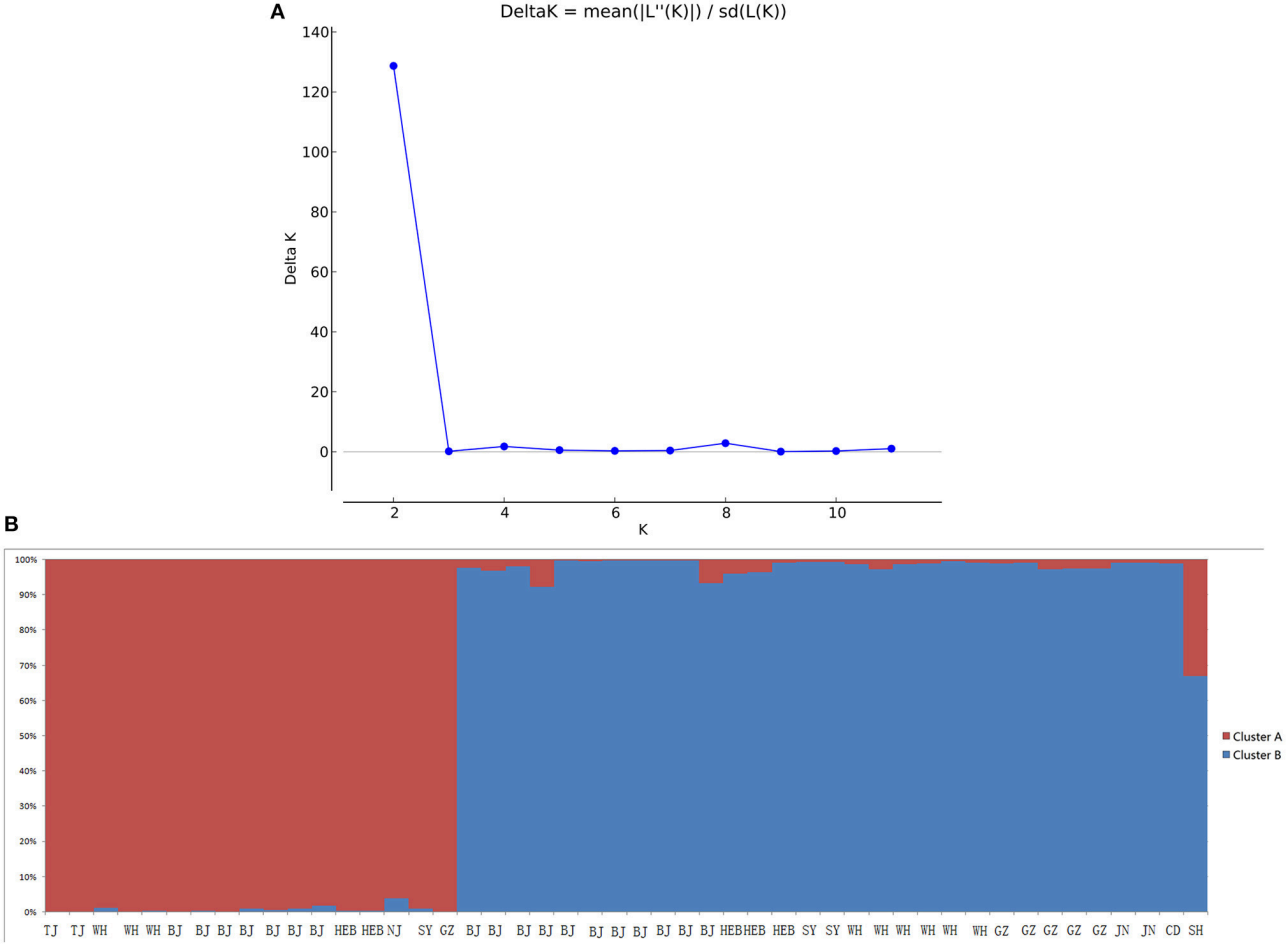
STRUCTURE analyses of 48 *C. krusei* strains. **(A)** STRUCTURE analysis estimates that the optimal predicted number of populations K for our set of genotypes is two. **(B)** Bayesian estimation of the population structure of *C. krusei* by STRUCTURE. Each vertical bar represents one individual and is partitioned into colored segments that represent the individual's estimated membership fractions in K clusters.

**Figure 3:**
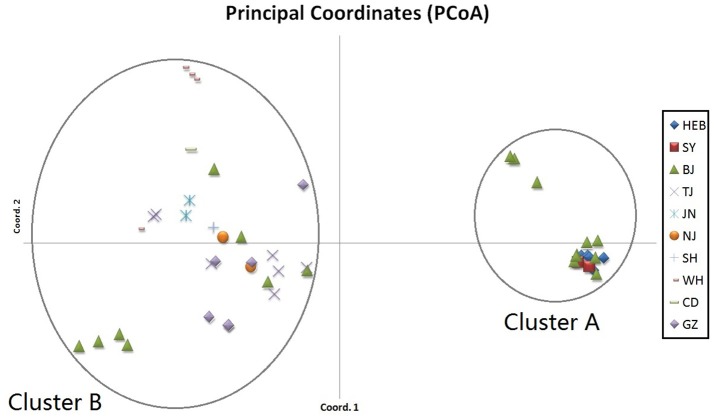
Results of principal coordinate analysis (PCoA) of *C. krusei* clusters. Using estimates of Nei's unbiased genetic distance supports 2 main subgroups, which corresponded to the 2 clusters divided by STRUCTURE software.

**Figure 4:**
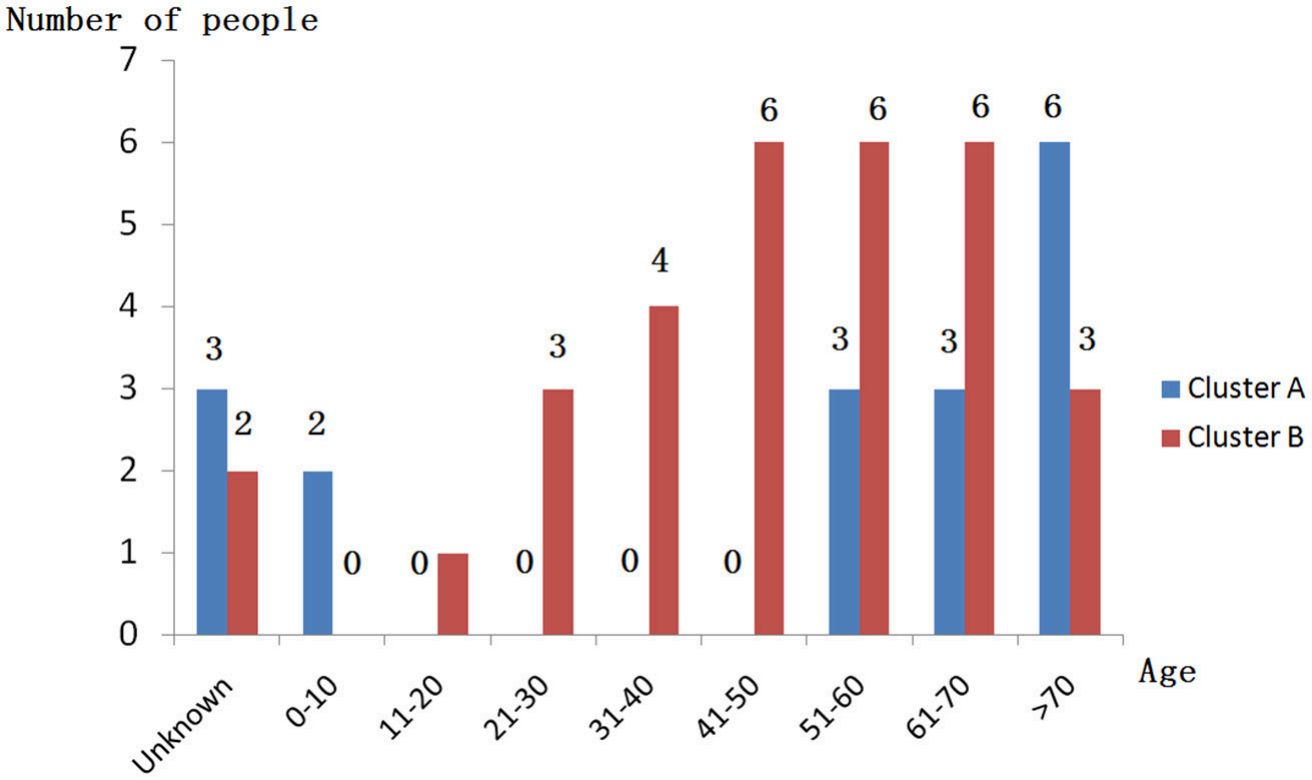
Age distribution of patients isolated from strains of two clusters.

**Figure 5:**
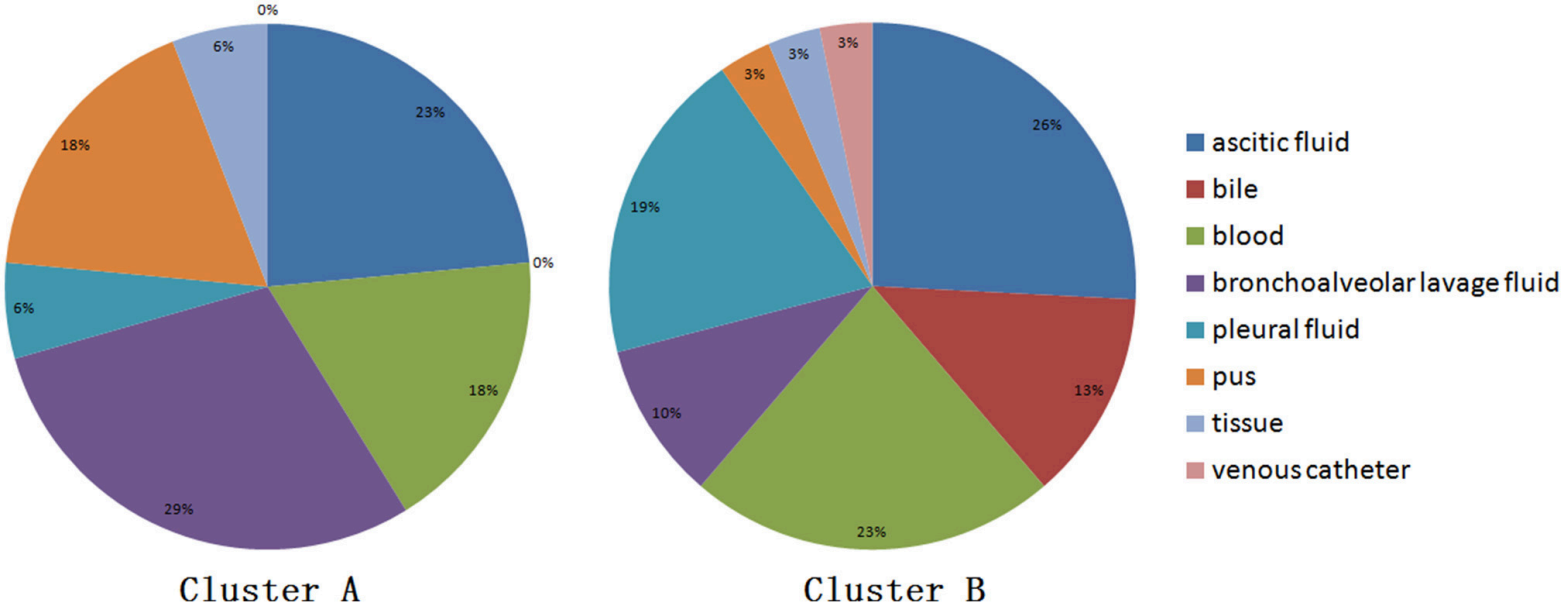
Specimen type isolated from strain of two clusters.

When performing STRUCTURE analyses, the clustering level *K* = 2 yielded the largest delta-K value (Figure 2A). At *K* = 2, individual isolates could be assigned to two clusters (Figure 2B). Cluster A included 17 strains and cluster B 31 strains. There was no clear relationship between cluster patterns and geographical source of the isolates, and between cluster patterns and disease clinical manifestations. The F_ST_ between the two clusters was 0.188 (*P* < 0.01). The principal coordinate analysis (PCoA) supported the result of the STRUCTURE analyses (Figure 3), suggesting that the population of *C. krusei* was divided into two lineages. The PCoA also suggested that two lineages would both consist of strains from different geographical origins and clinical manifestations of disease. However, if patients from whom the isolates were obtained is considered, there appears to be some differences between the two clusters. The hosts of Cluster A strains were mainly children younger than 10 years old or aged people older than 50 years old. In contrast, the hosts of Cluster B covered almost all age groups (Figure 4). Furthermore, the specimen types of Cluster B are also more complex than Cluster A (Figure 5).

The genetic diversity of *C. krusei* was assessed by Shannon's Information Index, Nei's unbiased gene diversity and allelic richness. These indices are shown in Table 2. Four indices (Mean number of effective alleles, Shannon's Information Index, Nei's unbiased gene diversity, allelic richness), all showed that there was a higher genetic diversity in cluster B.

**Table 2:** Genetic diversity of *Candida krusei* subgroups.

**Subgroup**	**Number of strains**	**N_A_^a^**	**N_e_^b^**	**I^c^**	**H_S_^d^**	**AR^e^**
Cluster A	17	2.545	1.865	0.645	0.416	2.527
Cluster B	31	7.667	4.186	1.583	0.737	6.659
Total	48	5.106	3.025	1.114	0.576	6.165

a Mean number of alleles;

b Mean number of effective alleles;

c Shannon's Information Index;

d Nei's unbiased gene diversity;

e *Allelic richness*.

### *In vitro* susceptibilities

**Figure 6:**
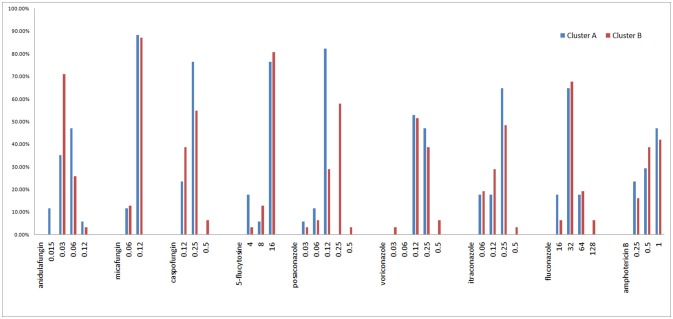
MIC range and proportion of two *C. krusei* clusters.

All isolates were intrinsically resistant to fluconazole (MICs ≥ 16 mg/L; Figure 6, Supplemental A). Of the other eight antifungal agents tested, all isolates were susceptible or of wild-type phenotype to voriconazole, itraconazole, posaconazole, anidulafungin, micafungin, 5-flucytosine, and amphotericin B. Only two of 48 isolates (4.2%) were interpreted as intermediate to caspofungin, both of which belonged to microsatellite cluster B, while the rest 95.8% (46/48) isolates remained susceptible to caspofungin (Figure 6, Supplemental A).

The Geometric mean (GM) MIC, MIC_50_, and MIC_90_ of the two clusters were generally similar, while the MIC range differed between the two clusters (Table 3, Figure 6). For most antifungal agents (6/9) (including caspofungin, posaconazole, voriconazole, itraconazole, fluconazole, amphotericin B), cluster B had a wider MIC range. It is worth noting that the highest MIC values of all 6 antifungal agents were confined to cluster B.

**Table 3:** GM MIC, MIC50, MIC90, and MIC range of *C.krusei* subgroups.

**Subgroup**		**Anidulafungin (μg/ml)**	**Micafungin (μg/ml)**	**Caspofungin (μg/ml)**	**5-Flucytosine (μg/ml)**	**Posaconazole (μg/ml)**	**Voriconazole (μg/ml)**	**Itraconazole (μg/ml)**	**Fluconazole (μg/ml)**	**Amphotericin B (μg/ml)**
Cluster A	GM MIC^a^	0.04	0.11	0.21	12.03	0.15	0.17	0.17	32.00	0.59
	MIC_50_^b^	0.06	0.12	0.25	16	0.25	0.12	0.25	32	0.5
	MIC_90_^c^	0.06	0.12	0.25	16	0.25	0.25	0.25	64	1
	MIC range	0.015–0.12	0.06–0.12	0.12–0.25	4–16	0.03–0.25	0.12–0.25	0.06–0.25	16–64	0.25–1
Cluster B	GM MIC	0.04	0.11	0.20	13.68	0.18	0.17	0.16	38.27	0.63
	MIC_50_	0.03	0.12	0.25	16	0.25	0.12	0.25	32	0.5
	MIC_90_	0.06	0.12	0.25	16	0.25	0.25	0.25	64	1
	MIC range	0.03–0.12	0.06–0.12	0.12–0.5	4–16	0.03–0.5	0.03–0.5	0.06–0.5	16–128	0.25–2
Overall	GM MIC	0.04	0.11	0.20	13.07	0.17	0.17	0.16	35.92	0.61
	MIC_50_	0.03	0.12	0.25	16	0.25	0.12	0.25	32	0.5
	MIC_90_	0.06	0.12	0.25	16	0.25	0.25	0.25	64	1
	MIC range	0.015–0.12	0.06–0.12	0.12–0.5	4–16	0.03–0.5	0.03–0.5	0.06–0.5	16–128	0.25–2

a Geometric mean Minimum inhibitory concentration;

b Mean minimal inhibitory concentrations against 50 percent of strains;

c *Mean minimal inhibitory concentrations against 90 percent of strains*.

## Discussion

The correlation between genetic diversity and adaptive capacity of the population has long been studied in the field of molecular ecology. For example, it was found that the *Arabidopsis thaliana* population with higher genetic diversity had better colonization success (Crawford and Whitney, 2010). In principle, the process of fungal infection and clinical manifestation of disease is also considered a colonization success. Unfortunately, very few studies have been done on fungal infections and drug-resistance from the perspective of population genetics. In this study, we carried out a comprehensive analysis of the population genetic features and drug resistance, and attempted to elucidate the drug resistance of *C. krusei* from an evolutionary perspective.

Although an important pathogenic fungal species, the population genetic parameters of *C. krusei* have remained largely unknown. In this study, a novel array of microsatellite markers was developed for molecular typing and population genetic analysis of the species. The discriminatory index of the new method (1.000) was slightly higher than MLST, which exhibited a discriminatory index of 0.998 (Jacobsen et al., 2007). It has been shown by Cuomo et al. (2017) that the genome of *C. krusei* is highly heterozygous, and this was also confirmed in the present study. For all microsatellite loci, there were some heterozygous individual isolates. As previously described in *Candida albicans*, a significant departure from Hardy-Weinberg equilibrium expectations was found (Sampaio et al., 2003). This finding supports the previous conclusions that reproduction in *C. krusei* is mainly clonal.

Based on the STRUCTURE software analysis, the *C. krusei* population in China appears to be divided into two clusters, a result which was also supported by PCoA. The two clusters showed no obvious differences with respect to geographical distribution of the isolates. These findings are very similar to those of another pathogenic fungus, *Trichophyton rubrum*, which was also divided into two clusters with similar geographical distributions of the clusters (Gong et al., 2016). For pathogenic fungi, it might be a common phenomenon that different clusters of the same organism co-exist in the same geographical locale. Dispersal of pathogenic fungi is generally affected by host activity. It is highly possible that *C. krusei* strains of different clusters existed in different geographical areas and were carried to the same geographical area by hosts including humans. When the survival capacity of different clusters is similar, it indicates that the different clusters co-existed in the same area. However, there were significant differences in host age between the two clusters, which might suggest that cluster B strains have a higher pathogenicity. Moreover, the type of specimens in Cluster B were also more complicated and seemed to confirm this. However, this is only a hypothesis, and more work needs to be done to demonstrate these findings in animal infection model experiments.

Cluster B had a higher genetic diversity which suggests a better adaptive capacity for survival in challenging conditions. As previously mentioned, the population with a higher genetic diversity is more likely to develop antimicrobial drug resistance. In our study, cluster B had a wider MIC range for 6 antifungal drugs, although there was no obvious difference in GM MIC, MIC_50_, and MIC_90_ between the two clusters. Specifically, the strains with the highest MIC value were either in the current group B (including 6 drugs: caspofungin, posaconazole, voriconazole, itraconazole, fluconazole, and amphotericin B), or both in group A and group B (including 3 drugs: anidulafungin, micafungin, and 5-flucytosine). Meanwhile, there was no strain with the highest MIC value was observed in Cluster A. These findings suggest that the population with higher genetic diversity may have more diverse phenotypes, including drug resistance. When subjected to selective pressure from antifungal drugs, cluster B might have a better adaptive capacity, and thus would be more likely to develop drug resistance. This suggests that the *C. krusei* population or lineage with higher genetic diversity needs more attention in terms of fungal drug resistance.

In conclusion, *C. krusei* was divided into two clusters by novel high-resolution microsatellite markers. The cluster with higher genetic diversity had wider MIC ranges for six antifungal agents, and the highest MIC values of the six antifungal agents belonged to the cluster of higher genetic diversity. It is plausible that the *C. krusei* cluster with higher genetic diversity might have better adaptive capacity when under the selection pressure of antifungal agents.

## Author contributions

JZ and Y-CX designed the experiments. JG, MX, HW, FZ, LH, and WW collected the samples and performed the experiments. JG, MX, TK, and YW analyzed data. JG, MX, and TK wrote the manuscript. All authors read and approved the final manuscript.

### Conflict of interest statement

The authors declare that the research was conducted in the absence of any commercial or financial relationships that could be construed as a potential conflict of interest.
